# Convergent validity and ecological validity of the Test of Visual Perceptual Skills-4th Edition in people with schizophrenia

**DOI:** 10.1192/j.eurpsy.2022.1993

**Published:** 2022-09-01

**Authors:** E.-C. Chiu, Y.-C. Lee, S.-C. Lee

**Affiliations:** 1 National Taipei University of Nursing and Health Sciences, Long-term Care, Taipei City, Taiwan; 2 College of Medical and Health Science, Asia University, Occupational Therapy, Taichung, Taiwan; 3 Taipei City Psychiatric Center, Taipei City Hospital Songde Branch, Occupational Therapy, Taipei City, Taiwan

**Keywords:** ecological validity, visual perception, schizophrénia, Convergent validity

## Abstract

**Introduction:**

Visual perceptual deficit commonly occurs in people with schizophrenia. The Test of Visual Perceptual Skills-4th Edition (TVPS-4) is a motor-free visual perceptual measure, which includes seven subtests: visual discrimination, visual memory, spatial relationships, form constancy, sequential memory, visual figure-ground, and visual closure. However, convergent validity and ecological validity of the TVPS-4 is largely unknown, which limits its use in clinical and research settings.

**Objectives:**

The purpose of this study was to examine convergent validity and ecological validity in people with schizophrenia.

**Methods:**

Ninety-nine people with schizophrenia were assessed the TVPS-4, the Mini Mental State Examination (MMSE), the Behavioral Assessment of the Dysexecutive Syndrome (BADS), and the Activities of Daily Living Rating Scale III (ADLRS-III). To evaluate convergent validity, Pearson’s *r* were calculated among the TVPS-4 and two cognitive measures (the MMSE and the BADS). To evaluate ecological validity, we computed correlation (*r*) between the TVPS-4 and the ADLRS-III.

**Results:**

The TVPS-4 total score showed moderate correlations with two cognitive measures (*r*=0.65-0.70). The seven TVSP-4 domains revealed moderate correlations with two cognitive measures (*r*=0.42-0.69). Moderate correlation (*r*=0.56) was found between the TVPS-4 total score and the ADLRS-III. Moderate to high correlations (*r*=0.33-0.61) were noticed among the seven TVPS-4 domains and the ADLRS-III.

**Conclusions:**

The TVPS-4 has good convergent validity and ecological validity in people with schizophrenia. The multiple domains of the TVPS-4 are useful to comprehensively identify visual perception deficits in people with schizophrenia. The TVPS-4 can adequately exhibit the degree of living independently in people with schizophrenia.

**Disclosure:**

No significant relationships.

